# High-yield production and functional analysis of novel rhizobial-type glutaminase-free l-asparaginase from *Paenibacillus thiaminolyticus*

**DOI:** 10.1007/s12223-025-01296-y

**Published:** 2025-07-11

**Authors:** Tomáš Podzimek, Karolína Loužecká, Veronika Urbánková, Jan Beránek, Petra Lipovová, Eva Benešová

**Affiliations:** Department of Biochemistry and Microbiology, University of Chemistry and Technology, Technická 5, 166 28 Prague 6, Czech Republic

**Keywords:** *Paenibacillus thiaminolyticus*, Asparaginase, Acute lymphoblastic leukemia, Acrylamide mitigation, Food industry, Biosensors

## Abstract

l-Asparaginases are enzymes known for decades due to their use in medicine for the treatment of acute lymphoblastic leukemia. Recently, they have also found application in the food industry, and other possibilities are emerging in the treatment of infectious diseases or in the design of biosensors. For this reason, an ongoing effort has been made to find and characterize new enzymes with properties suitable for these specific applications. In this work, l-asparaginase from *Paenibacillus thiaminolyticus* (isoenzyme 1) belonging to the least explored group of l-asparaginases derived from l-asparaginase from *Rhizobium etli* was recombinantly produced with high yields (335 mg per L of culture medium) in *E. coli* cells and characterized: *K*_*M*_ = (26 ± 8) mmol L^−1^, pH_opt_ = 10.3, without l-glutaminase and urease activity. A probable oligomeric structure (homodimer) was derived by computer modeling and confirmed by gel chromatography experiments. The results of this work extend the current limited knowledge about the poorly described class of *R. etli* l-asparaginases. Moreover, this l-asparaginase exhibits suitable properties for use in biosensor construction because of the high yields during recombinant production, *K*_*M*_ value, stability, and absence of l-glutaminase activity.

**Supplementary Information:**

The online version contains supplementary material available at https://doi.org/10.1007/s12223-025-01296-y.

## Introduction

l-Asparaginases (EC 3.5.1.1) represent a broad group of enzymes characterized by their ability to hydrolyze l-asparagine to form aspartic acid and ammonia (Nunes et al. 2020). l-Asparaginases are divided into three classes, according to their amino acid sequence, structure, and biochemical properties. A number of representatives of classes 1 (bacterial) and 2 (plant) are described, characterized, and even used in medicine or the food industry, while our knowledge about class 3 (rhizobial) asparaginases is still very limited and only a few representatives of class 3, *Rhizobium etli*-type l-asparaginases, have been characterized so far (Loch and Jaskolski 2021). Those l-asparaginases come from *Paenibacillus barengoltzii*, *Synechocystis* sp*.*, *Nocardiopsis alba*, *Streptomyces griseus*, and *Rhizobium etli* (ReaV and ReAIV). Therefore, some effort should be made to broaden the knowledge about this class and to suggest whether these asparaginases would also find application in biotechnological practice.

The potential of l-asparaginases in the therapy of cancer diseases was discovered at the beginning of the second half of the twentieth century (Kidd 1953; Broome 1963). The possibility of l-asparaginases application in the treatment of acute lymphoblastic leukemia and other lymphoproliferative disorders results from the fact that leukemic cells are not able to synthesize l-asparagine, which they need for their rapid growth in sufficient amounts (because of low levels of asparagine synthetase) and depend on its external supply from the blood (Egler et al. 2016; Shakambari et al. 2019; Castro et al. 2021). Therefore, deficiency of l-asparagine in the blood caused by administration of l-asparaginase will result in the death of cancer cells (Shakambari et al. 2019). In contrast, healthy cells can meet their l-asparagine demands through the catalytic activity of l-asparagine synthetase and, therefore, a decrease in the concentration of this proteinogenic amino acid in serum is not fatal for them (Maese and Rau 2022). The discovery of these unique abilities of l-asparaginases led to their introduction into the standard treatment procedure and significantly increased the success of the treatment of pediatric patients suffering from acute lymphoblastic leukemia (Egler et al. 2016).

Due to the events of 1997, when a large amount of acrylamide was released into nature during a new railroad tunnel construction, a new era of l-asparaginases application started, this time in the food industry (Reynolds 2002). Different studies confirmed the presence of acrylamide, a neurotoxic and potentially carcinogenic substance, in starch-rich food processed at high temperatures all over the world (independent of the leak in 1997) and organizations such as WHO (World Health Organization), EFSA (European Food Safety Authority), or FAO (Food and Agriculture Organization) agreed to the need to monitor and regulate its concentration in food (Reynolds 2002; Castro et al. 2021; Abedi et al. 2023). Nowadays, there exist many different approaches to reduce the concentration of acrylamide in final food products, but most of them negatively influence the product characteristics, as, e.g., texture, flavour, and nutritional parameters. The use of l-asparaginases, which can cleave the main precursor of acrylamide formation (l-asparagine), is an alternative to these methods that do not suffer from their shortcomings (Abedi et al. 2023). Currently, l-asparaginases originating from *Aspergillus niger* and *Aspergillus oryzae* are commercially used (Abedi et al. 2023) to decrease the l-asparagine content in the raw materials of risk foods.

Since then, l-asparaginases have been the main interest of many scientists, and new possibilities of their application are gradually appearing. In the field of medicine, it has been proven that medicinal products containing l-asparaginases could be used in the treatment of some infectious diseases, e.g., caused by the pathogenic bacterium group A *Streptococcus* (Baruch et al. 2014) or could have a potential in the treatment of autoimmune disorders as, e.g., different types of arthritis (Reiff et al. 2001; Vimal and Kumar 2018; Hosseini et al. 2024). Biosensors containing l-asparaginases could be used for the determination or monitoring of l-asparagine concentration in blood during cancer treatment or in raw materials for food production (Nunes et al. 2021). However, to our best knowledge, no commercial l-asparaginase without l-glutaminase activity is available, which is crucial for the precise determination of l-asparagine level.

Although l-asparaginases from many different organisms, including mammals, plants, and microorganisms were described, new sources of l-asparaginases are still being sought, which would surpass the enzymes currently used in terms of their properties (Shakambari et al. 2019). This article presents a new l-asparaginase (ASN339Pt iso 1) originating from the bacterium *Paenibacillus thiaminolyticus*, which was prepared and characterized in the form of a fusion protein with a polyhistidine tag. Important characteristics that determine the biotechnological potential of this l-asparaginase compared to commercially available l-asparaginases are simple production providing high yields, suitable biochemical properties, and the absence of l-glutaminase activity. At the same time, this work extends the number of characterized l-asparaginases from the rhizobial class and confirms the importance of their further study.

## Materials and methods

### Microorganisms used

The bacterial strain *E. coli* DH5α (GibcoBRL, Carlsbad, CA, USA) was used for the isolation of the plasmid pET16b-ASN339Pt iso 1. Bacterial strains *E. coli* BL21 (DE3) (Novagen, Madison, WI, USA) and *E. coli* Lemo21 (DE3) (New England Biolabs) were used for enzyme production.

The bacteria *Paenibacillus thiaminolyticus* was obtained from the Czech Collection of Microorganisms in Brno (CCM 3599) and used as a source of DNA.

### Construction and isolation of plasmids pET16b–ASN339Pt iso 1 and pET16b–ASN339Pt iso 1 (K52H)

The plasmid pET16b-ASN339Pt was constructed by ligation of the linearized vector pET16b and the ASN339Pt iso 1 l-asparaginase gene (coding a protein 339 amino acids long) using Gibson Assembly (Gibson Assembly®Master Mix, NEB). Both the nucleotide and amino acid sequences of ASN339Pt iso 1 are depicted in Online Resource 1. The vector was linearized by PCR (Q5®High-Fidelity 2 × Master Mix, NEB) using primers listed in Table 1. The gene was amplified from genomic DNA isolated from the culture of *Paenibacillus thiaminolyticus* (GenElute™ Bacterial Genomic DNA Kit) cultivated in liquid Luria–Bertani (LB) medium for 16 h at 30 °C in a platform shaker (250 RPM) by PCR using primers listed in Table 1. The correct insertion of the gene into the vector was confirmed by sequencing. The PCR-based (Q5®High-Fidelity 2 × Master Mix, NEB) site-directed mutagenesis was used to change the Lys52 codon to His52 codon, resulting in the plasmid pET16b-ASN339Pt iso 1 (K52H). The sequences of the used primers are listed in Table 1. The plasmid pET16b-ASN339Pt iso 1 (K52H) was then assembled using Gibson Assembly, and the correct codon substitution in the plasmid was confirmed by sequencing.

**Table 1:** Primers used for both amplification of the l-asparaginase gene and linearization of the vector pET16b, and primers used for PCR-based site-directed mutagenesis (K52H), the introduced mutation is underlined

Name	Sequence
l-asparaginase forward	5′ GATCCTCGAGTTACTTTTTAACCCACTTC 3´
l-asparaginase reverse	5′ TCATATGATGGTAGACTTGGGTAAAGTGTTAG 3′
pET16b forward	5′ GTCTACCATCATATGACGACCTTCGATATG 3′
pET16b reverse	5′ AGTAACTCGAGGATCCGGCTGCTAAC 3′
K52H fwd	5′ ATCATCGCATCCGGTTCAGGCGCTGCCCG 3′
K52H rev	5′ GAACCGGATGCGATGATGAACGCATGAAGC 3′

### Production of recombinant ASN339Pt iso 1

Two different commercially available production strains of *E. coli* were used for the production of recombinant enzyme ASN339Pt iso 1. Production in the commonly used strain *E. coli* BL21 (DE3), using simple IPTG induction of recombinant protein production, was compared to production in the strain of *E. coli* Lemo21 (DE3), which is designed to slow the velocity of the protein production using rhamnose as a modulator (in addition to IPTG induction). Cells were transformed by the plasmid pET16bASN339Pt iso 1 and cultivated overnight on an LB agar plate supplied with ampicillin (LBA medium, final concentration of ampicillin 100 μg mL^−1^) at 37 °C. The next day, a single colony was inoculated in 10 mL of LBA medium. After overnight cultivation at 37 °C/120 RPM, the obtained culture was used for a 1% inoculation of the final volume of LBA medium (200 mL). In the case of *E. coli* Lemo21 (DE3), LBA medium was supplemented from the beginning with rhamnose in concentrations of 50, 100, or 500 μmol L^−1^. The cultivation was carried out at 37 °C/200 RPM until OD ÷ 0.5 was reached, and then the production of the recombinant protein was induced by isopropyl β-d-thiogalactopyranoside (IPTG) at a final concentration of 0.3 mmol L^−1^ in both cases. Cells were harvested after 4 or 10 h after induction or after an overnight post-induction cultivation, centrifuged, and the pellet was stored at − 20 °C. When both strains were cultivated in autoinduction medium (Overnight Express™ Instant LB Medium, EMD Millipore), no IPTG was added. The cultivation was carried out at 37 °C for either 10 h or overnight. After the cultivation, the cells were pelleted and stored at − 20 °C.

### Isolation and purification of ASN339Pt iso 1

The cell pellet obtained was resuspended in 100 mM Tris buffer, pH 9 containing 150 mM NaCl. Cells were disintegrated by the OneShot disruptor (Constant Systems Ltd., GB) at 2.7 kbar. The supernatant was obtained by centrifugation at 30,000 g/20 min/4 °C and applied to Ni–NTA agarose (QIAGEN). The column was washed twice with 100 mM Tris buffer, pH 9 containing 150 mM NaCl and 10 mM imidazole, and twice with the same buffer containing 40 mM imidazole. l-Asparaginase was eluted with 50 mM Tris buffer, pH 9 containing 150 mM NaCl and 250 mM imidazole. Fractions with active l-asparaginase were desalted using a PD-10 column (GE Healthcare) and stored at − 20 °C. The concentration of proteins was determined by the Pierce™ BCA Protein Assay Kit according to the manufacturer’s instructions. The enzyme was designated as ASN339Pt iso 1 (l-asparaginase containing 339 amino acids, originating from *P. thiaminolyticus*, isoenzyme 1).

### Determination of ASN339Pt iso 1 activity

The reaction mixtures consisted of 290 μl of 10 mM l-asparagine (Roth) dissolved in 100 mM Tris pH 9.0 and 10 μl of enzyme solution, and the activity assay was carried out at 37 °C for 10 min. The reaction was stopped by adding 15 μL of 1.5 M trichloroacetic acid. Then, 36 μL of Nessler’s reagent (Supelco) were added and the mixture was incubated at laboratory temperature for 10 min. The absorbance was measured at 436 nm. One U was defined as the amount of ASN339Pt that releases 1 μmol of ammonia per minute.

### Biochemical characterization of ASN339Pt iso 1

#### Effect of pH

The dependence of activity on pH was measured in Britton-Robinson buffers with a pH ranging from 8.1 to 11.9.

#### Effect of temperature

The effect of temperature on activity was tested in the range from 15 to 60 °C.

#### Kinetic measurement

For the measurement of the effect of substrate concentration on l-asparaginase activity, reaction mixtures containing l-asparagine at concentrations ranging from 1.9 to 145 mmol L^−1^ were used. The data was evaluated using the substrate inhibition model for one additional substrate molecule (Eq. 1) and the modified substrate inhibition model, which is represented by Eq. (2) (Bapiro et al., 2018).

1 $${v}_{0}=\frac{{V}_{lim}\bullet \left[S\right]}{{K}_{m}+\left[S\right]\bullet \left(1+\frac{\left[S\right]}{{K}_{i}}\right)}$$

2 $${v}_{0}=\frac{{V}_{lim}\bullet \left[S\right]}{{K}_{m}+\left[S\right]\bullet \left(1+\frac{{\left[S\right]}^{n}}{{{K}_{i}}^{n}}\right)}$$

#### l-Glutaminase and urease activity of ASN339Pt iso 1

The reaction mixture was the same for both substrates as for l-asparagine and contained 30 and 24 mmol L^−1^ of l-glutamine or urea, respectively, dissolved in 100 mM Tris pH 9.0. Reactions with l-glutamine and urea were carried out for 90 h and 20 h at 37 °C, respectively. Urease activity was determined using Nessler’s method, while l-glutaminase activity was determined by thin layer chromatography (TLC) on silica gel/TLC-card (Fluka). The samples were separated on the TLC card using a phenol:water solution (2:1, v/v), stained with 0.2% ninhydrin in acetone, and the color was developed by heating with a hair dryer. l-Glutamine (Roth) and l-glutamic acid (Merck) were used as standards.

#### Effect of a high concentration of l-aspartic acid and NH_4_^+^

l-Aspartic acid was added to the reaction mixture at concentrations of 0.1 to 10 mmol L^−1^. Ammonium sulfate was added to the reaction mixture at concentrations of 30 to 380 μmol L^−1^. In both experiments, the reaction was carried out for 10 min at 37 °C.

#### Temperature stability of ASN339Pt iso 1 over time

ASN339Pt iso 1 was incubated at 25 °C, 37 °C, and 55 °C for 22 days, 72 h, and 4 h, respectively. An aliquot of 10 μl was taken at intervals, and the activity assay was performed.

#### Effect of NaCl

The effect of NaCl on ASN339Pt iso 1 activity was tested in the range of 0 to 500 mmol L^−1^ NaCl in the reaction mixture.

### The determination of oligomeric state of ASN339Pt iso 1

Size exclusion chromatography was used for the determination of *M*_*w*_ of native ASN339Pt iso 1. The sample was separated in the column HiPrep™ 16/60 Sephacryl S-100 HR (GE Healthcare) under these conditions: flowrate 0.5 mL min^−1^, 50 mM phosphate (K^+^) buffer, pH 8.0. The dead volume of the column was determined to be 42 mL using aldolase (160 kDa). The following proteins were used as standards: lysozyme (14 kDa), amylase (55 kDa), and aldolase (160 kDa).

### Prediction of the 3D structure and oligomeric state of ASN339Pt iso 1

The 3D structure of ASN339Pt iso 1 was predicted using AlphaFold 3 server (Abramson et al. 2024) based on the sequence of the gene for ASN339Pt iso 1. The structure was predicted four times in total for systems with one to four subunits. The expected position error of the predicted structure was observed between each subunit in multimeric systems to evaluate the likelihood of such a mutual position of enzyme subunits.

### Effect of zinc on ASN339Pt iso 1 activity

The effect of zinc was tested after application of the chelating agent ethylenediaminetetraacetic acid (EDTA). ASN339Pt iso 1 (0.3 mg) was incubated in 10 mM EDTA for 2 h on ice. The enzyme was then dialyzed overnight against 100 mM Tris buffer pH 9.0 at 10 °C and treated with the addition of ZnCl_2_ to final concentrations from 10 to 1000 μmol L^−1^. A dialyzed enzyme sample (0.3 mg) without EDTA treatment was also prepared, which served as a control for the effect of dialysis on the enzyme itself. The activities of both enzyme samples were determined as described above.

## Results

### Protein sequence of ASN339Pt iso 1

**Fig. 1:**
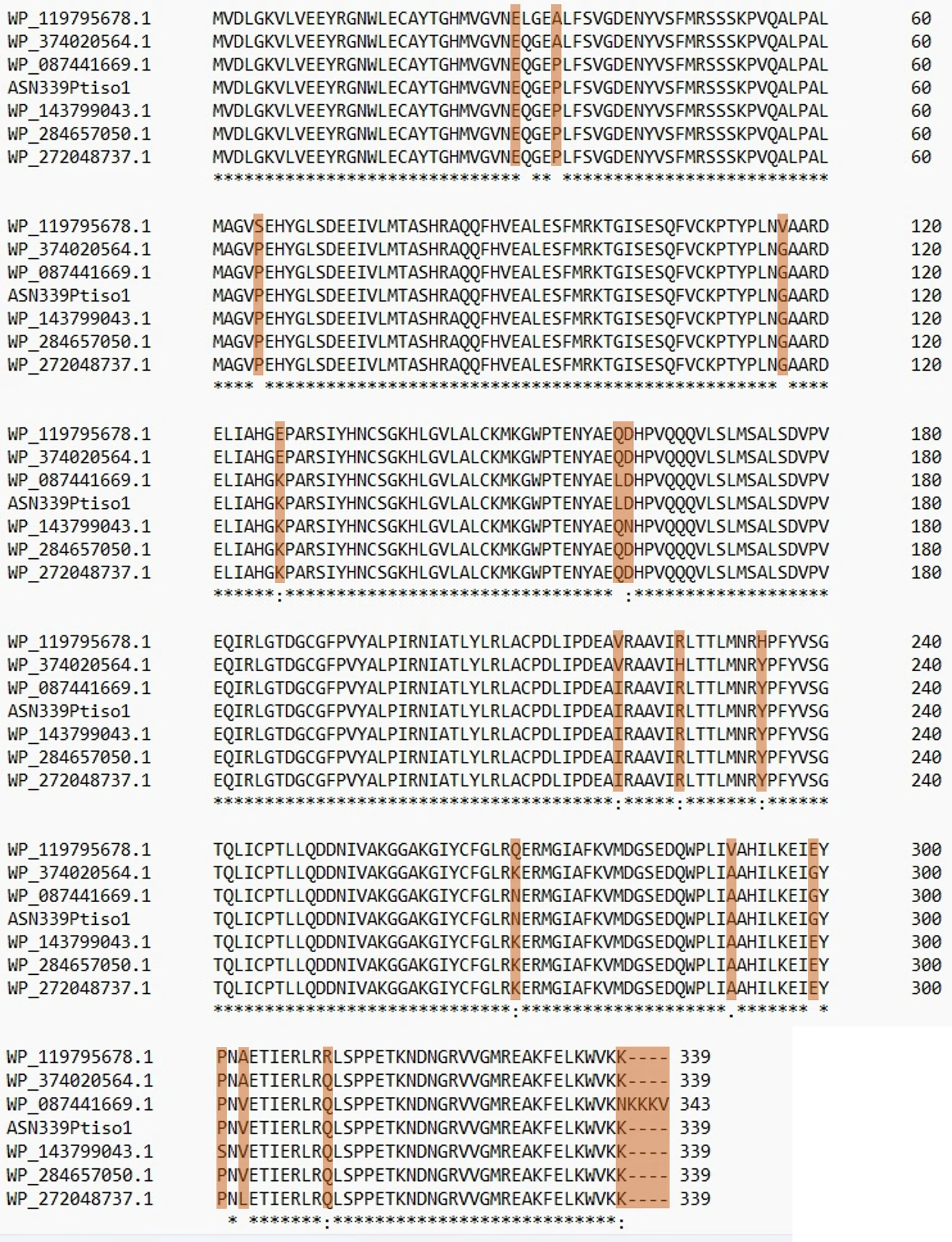
Alignment of the amino acid sequence ASN339Pt iso 1 with WP_284657050.1, WP_119795678.1, WP_272048737.1, WP_143799043.1, WP_374020564.1, and WP_087441669.1. All sequences refer to l-asparaginase from *P. thiaminolyticus*. Amino acids that differ in sequences are indicated by color. The alignment was made in CLUSTALW

Currently, we can conclude that the databases (searched by PROTEIN BLAST, nonredundant protein sequences (nr)) contain five amino acid sequences of l-asparaginases from *P. thiaminolyticus* of the same length (339 amino acids, WP_119795678.1, WP_284657050.1, WP_272048737.1 WP_374020564.1, and WP_143799043.1) and one sequence that is four amino acids longer (343 amino acids, WP_087441669.1). The alignment of these sequences is shown in Fig. 1, where it is visible that the sequences differ in several amino acids.

The nucleotide sequence of l-asparaginase ASN339Pt iso 1 obtained from genomic DNA of *P. thiaminolyticus* (CCM 3599) was sequenced, translated into amino acid sequence, and evaluated with the PROTEIN BLAST program. This sequence did not correspond to any of the sequences found in databases (Fig. 1). Using this sequence, l-asparaginase was recombinantly produced and characterized. This l-asparaginase should not be confused with the second isoenzyme present in *P. thiaminolyticus* (WP_087440700.1), which is not the subject of this scientific article.

### Recombinant production of ASN339Pt iso 1 in *E. coli* BL21 (DE3) and Lemo21 (DE3)

**Fig. 2:**
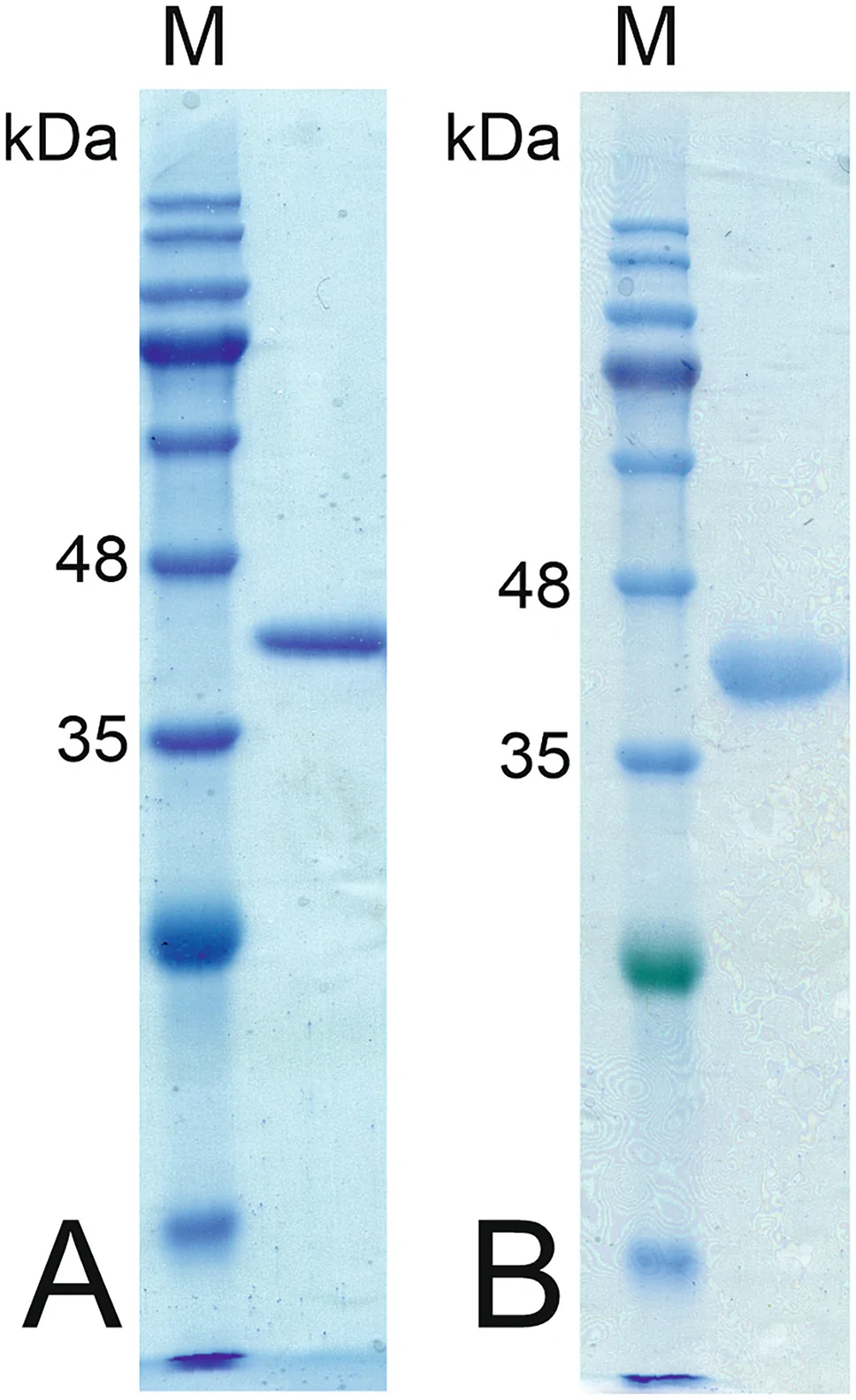
SDS-PAGE analysis of ASN339Pt iso 1 (40.3 kDa) after purification in the column containing Ni–NTA agarose. **A** Production in *E. coli* BL21 (DE3) in LB medium, 4 h of induction with IPTG. **B** Production in *E. coli* Lemo21 (DE3) in LB medium with 100 μmol L^−1^ of rhamnose, 4 h after induction with IPTG. M – BlueRay Prestained Protein Marker (10–180 kDa)

The recombinant protein ASN339Pt iso 1 was produced as a fusion protein with a histidine tag located at the N-terminal part of the amino acid sequence. Production of ASN339Pt iso 1 was tested in two different production systems, i.e., *E. coli* strains BL21 (DE3) and Lemo21 (DE3) under different conditions (Table 2), which had a significant impact on the final yield. Highly purified recombinant enzyme with a molecular weight of 40.3 kDa (including histidine tag) was obtained from the production in both used *E. coli* strains (Fig. 2). The best production yield of the highly purified enzyme obtained after production in *E. coli* BL21 (DE3) was approximately 150 mg L^−1^ (strain cultured overnight in autoinduction medium). However, the final yield of the purified enzyme after production in the *E. coli* Lemo21 (DE3) strain reached approximately 330 mg per L of culture medium. This yield was achieved at two different cultivation conditions: (a) LB medium, 4 h of cultivation after induction, 100 μM rhamnose and (b) autoinduction medium, overnight cultivation, 500 μM rhamnose (Table 2).

**Table 2:** Yields of purified ASN339Pt iso 1 after cultivation under different conditions

Medium, time^●^	Production strain	Effector	Final yield per L of cultivation medium
LB, 4 h	*E. coli* BL21 (DE3)	300 μmol L^−1^ IPTG	110 mg
LB, 4 h	*E. coli* Lemo21 (DE3)	300 μmol L^−1^ IPTG, 50 μmol L^−1^ rhamnose	170 mg
LB, 4 h	*E. coli* Lemo21 (DE3)	300 μmol L^−1^ IPTG, 100 μmol L^−1^ rhamnose	337 mg
LB, 4 h	*E. coli* Lemo21 (DE3)	300 μmol L^−1^ IPTG, 500 μmol L^−1^ rhamnose	80 mg
Autoinduction, 10 h	*E. coli* BL21 (DE3)	-	130 mg
Autoinduction, 10 h	*E. coli* Lemo21 (DE3)	50 μmol L^−1^ rhamnose	145 mg
Autoinduction, 10 h	*E. coli* Lemo21 (DE3)	100 μmol L^−1^ rhamnose	110 mg
Autoinduction, 10 h	*E. coli* Lemo21 (DE3)	500 μmol L^−1^ rhamnose	146 mg
LB, overnight	*E. coli* BL21 (DE3)	300 μmol L^−1^ IPTG	1 mg
LB, overnight	*E. coli* Lemo21 (DE3)	300 μmol L^−1^ IPTG, 50 μmol L^−1^ rhamnose	59 mg
LB, overnight	*E. coli* Lemo21 (DE3)	300 μmol L^−1^ IPTG, 100 μmol L^−1^ rhamnose	11 mg
LB, overnight	*E. coli* Lemo21 (DE3)	300 μmol L^−1^ IPTG, 500 μmol L^−1^ rhamnose	110 mg
Autoinduction, overnight	*E. coli* BL21 (DE3)	-	150 mg
Autoinduction, overnight	*E. coli* Lemo21 (DE3)	50 μmol L^−1^ rhamnose	200 mg
Autoinduction, overnight	*E. coli* Lemo21 (DE3)	100 μmol L^−1^ rhamnose	180 mg
Autoinduction, overnight	*E. coli* Lemo21 (DE3)	500 μmol L^−1^ rhamnose	335 mg

●The time stated corresponds to the cultivation time since induction of recombinant protein production

### Biochemical characteristics of ASN339Pt iso 1

**Fig. 3:**
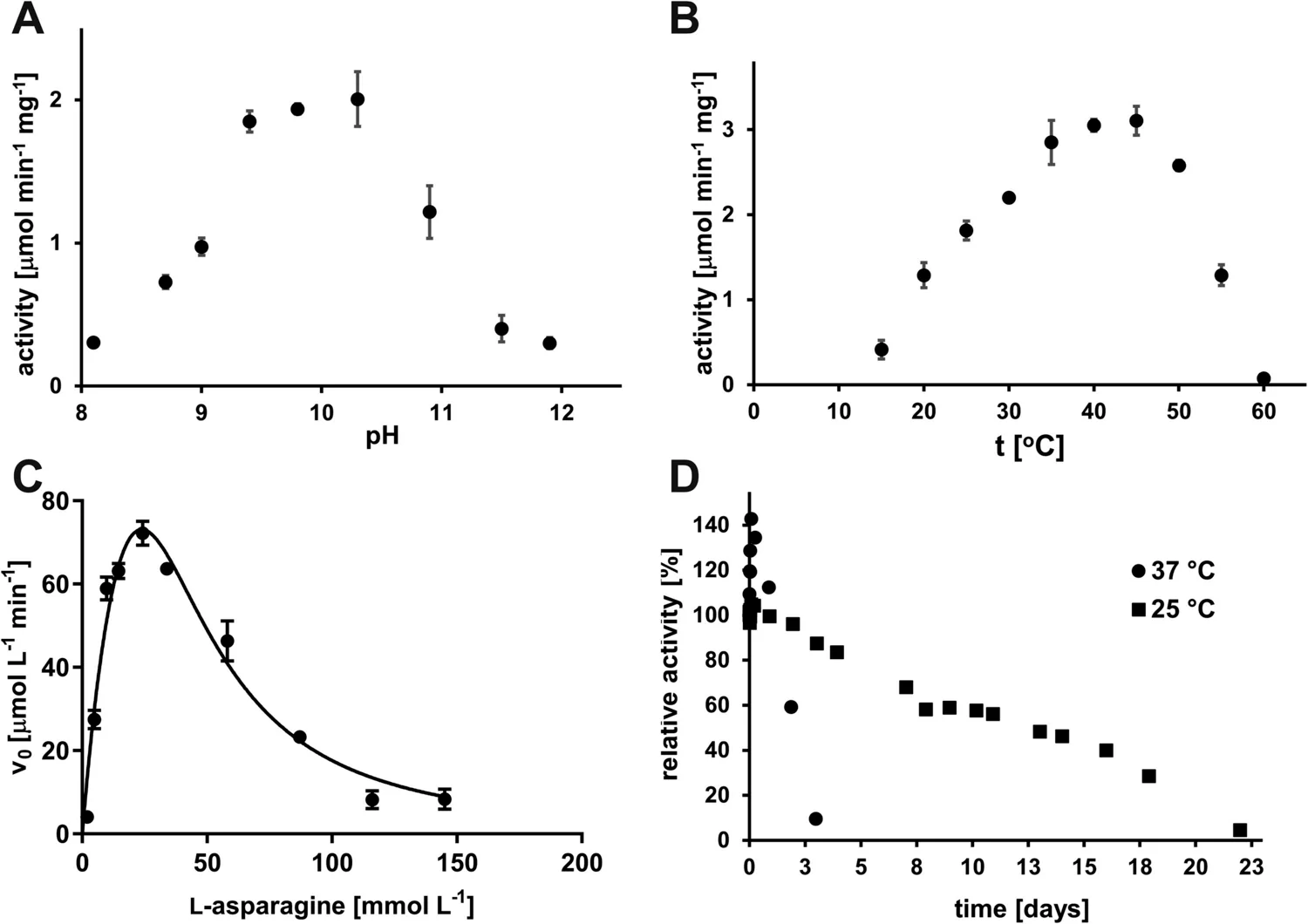
**A** The effect of pH on the activity of ASN339Pt iso 1. **B** The effect of temperature on the activity of ASN339Pt iso 1. **C** The effect of the concentration of l-asparagine on the activity of ASN339Pt iso 1. The points were fitted with a substrate inhibition model according to Bapiro et al. (2018) (Eq. (2)). The evaluation was performed in Graph Pad Prism 7. **D** The temperature stability of ASN339Pt iso 1. The plot shows the incubation of the enzyme at both 25 °C (■) and 37 °C (●), respectively

First, the pH optimum of the enzyme was determined. During the preparation of solutions, l-asparagine was found to significantly lower the pH of basic solutions. Therefore, the pH of the solution was always adjusted after the dissolution of l-asparagine. ASN339Pt iso 1 is active in the basic pH range, retaining approximately 15% of activity at both pH 8 and 12. The pH optimum is found approximately at pH 10.3 (Fig. 3A).

The temperature profile of ASN339Pt iso 1 is characterized by the maximal activity at 45 °C, by about 10% activity at 15 °C, and complete inactivation of the enzyme at temperatures of 60 °C and higher (Fig. 3B).

Temperature stability of ASN339Pt iso 1 was measured at 25 °C, 37 °C, and 55 °C. Relatively long-term stability was observed at 25 °C, when activity dropped to 50% after 13 days of incubation and was completely lost after 22 days of incubation (Fig. 3D). At 37 °C, the enzyme activity decreased approximately to 10% after 3 days (Fig. 3D). The overall activity was lost after 4 h at 55 °C (not shown here).

Kinetic measurements were performed to obtain the kinetic parameters *K*_*M*_ and *V*_max_. As can be seen in Fig. 3C, the behaviour of the enzyme does not correspond to the classical Michaelis–Menten kinetics, and the enzyme is probably inhibited by higher substrate concentrations. After application of the substrate inhibition model described in Eq. (1), the curve did not fit the experimental points well (plot not shown). Using a modified substrate inhibition model (Bapiro et al., 2018), the best fit of the curve with the highest coefficient of determination *R*^2^ was achieved when number 2 was substituted for the parameter *n* (Table 3). This means that two substrate molecules (in addition to the cleaved one) bind to the active site or any other site on the enzyme and inhibit the catalytic process. To be sure, the effect of aspartic acid and NH_4_^+^ (as the products of the reaction) on the activity of l-asparaginase was also measured; however, no inhibition was observed at all.

**Table 3:** Kinetic parameters obtained for ASN339Pt iso 1

Parameters	Results (according to Eq. (2))
*K*_*M*_	(26 ± 8) mmol L^−1^
*V*_max_	(194 ± 39) μmol L^−1^ min^−1^
*K*_*i*_	(32 ± 5) mmol L^−1^
*n*	2
*R*^2^	0.9583

The enzyme has also been tested for resistance to elevated salt concentration. In the presence of 150 mM NaCl, the enzyme still retains approximately 30% of its initial activity, and it is totally inhibited in the presence of 250 mM NaCl (Online Resource 2).

**Fig. 4:**
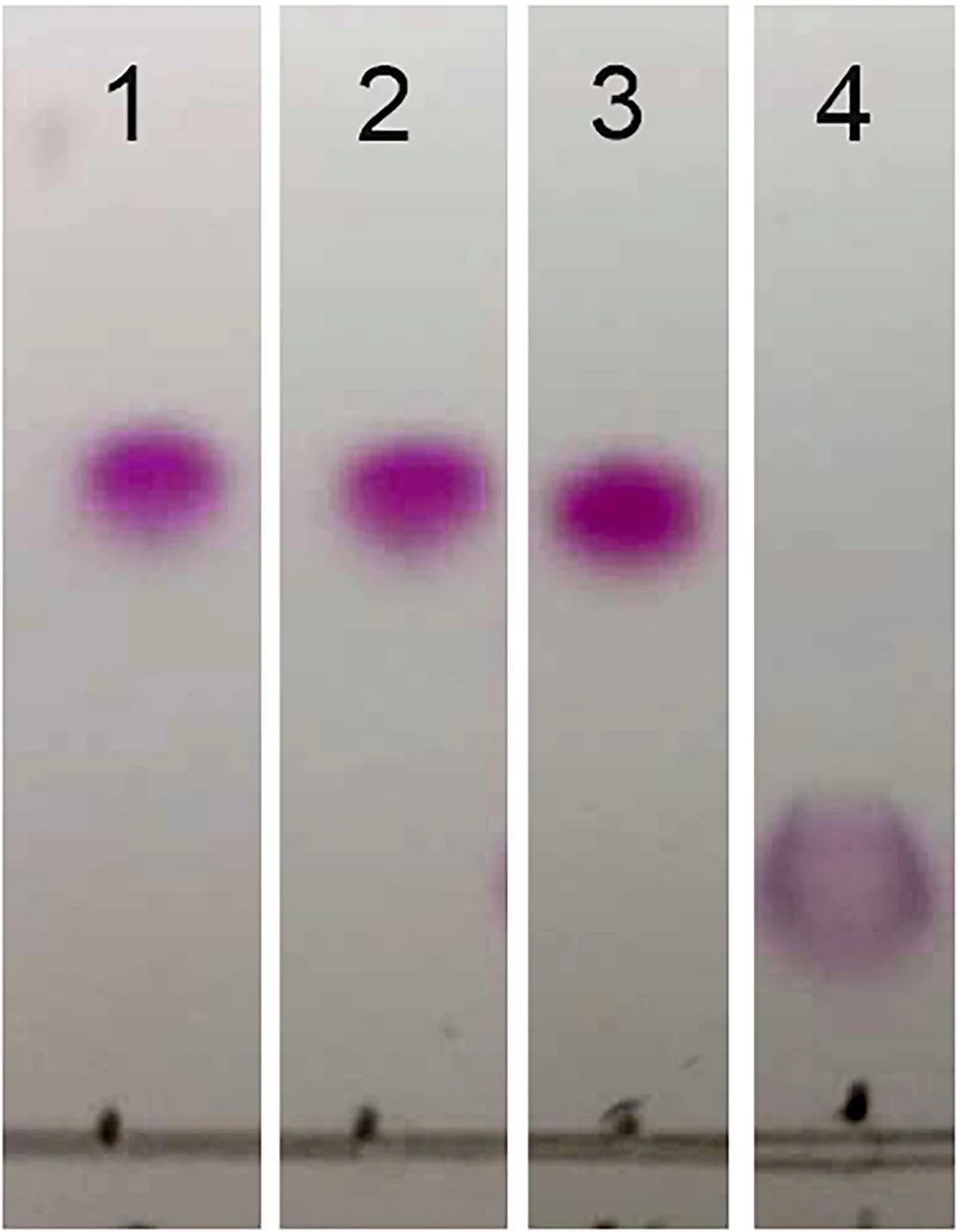
Determination of reaction products after l-glutamine cleavage by ASN339Pt iso 1 using TLC. **1**) Sample after 90 h reaction at 37 °C, **2**) reaction mixture without ASN339Pt iso 1 (30 nmol of l-Asn), **3**) l-glutamine standard (12 nmol), **4**) l-glutamate standard (12 nmol). The samples were stained with 0.2% ninhydrin in acetone, and the color developed by heating

The substrate specificity of the enzyme was verified in experiments using l-glutamine and urea as substrates. In the case of urea, no ammonia was detected as a product. Due to consistently high blank values when l-glutamine was used as a substrate, we chose TLC rather than the Nessler’s method for the detection of potential formation of l-glutamate by the action of l-asparaginase. As found out by Arii et al. (1999), l-glutamine is less stable in a high pH environment than l-asparagine. If we consider that Nessler’s reagent has pH more than 12, a significant amount of l-glutamine is non-enzymatically converted into pyroglutamate and ammonia. This ammonia then reacts with Nessler’s reagent, which leads to high absorption of the blank sample. The results showed that no l-glutamate was detected even after long-term reaction (Fig. 4, lane 1 compared to lane 4), which supports that ASN339Pt iso 1 is highly specific for l-asparagine.

### The oligomeric state of ASN339Pt iso 1 and its predicted 3D structure

l-Asparaginases are mostly multisubunit proteins, so an attempt was made to experimentally determine the number of subunits in ASN339Pt iso 1. The number of units in the native protein was determined using gel permeation chromatography, which revealed that it is a dimer with M_W_ about 80 kDa (Online Resource 3).

**Fig. 5:**
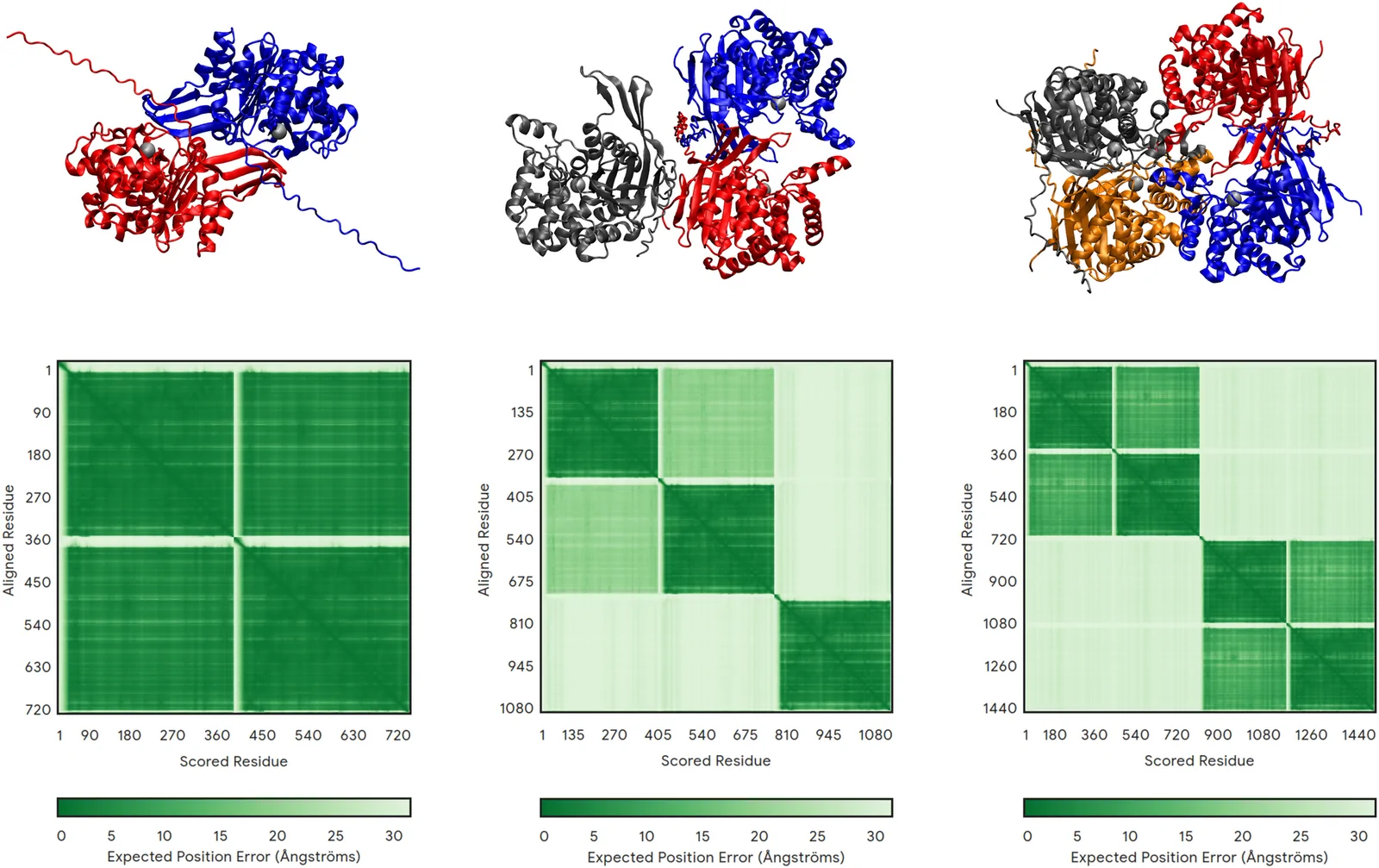
Structures of di-, tri-, and tetramer complexes of ASN339Pt iso 1 (respectively) predicted by the AlphaFold 3 server (top) and their expected relative position errors as predicted by AlphaFold 3

These results are also supported by AlphaFold 3, which predicts ASN339Pt iso1 dimer with a much lower intersubunit mutual position error than in the case of tri- or tetramer complexes (Fig. 5), suggesting that this protein forms two-subunit complexes at most. For the following work and comparison with the experimental structure, the two-subunit complex was used.

**Fig. 6:**
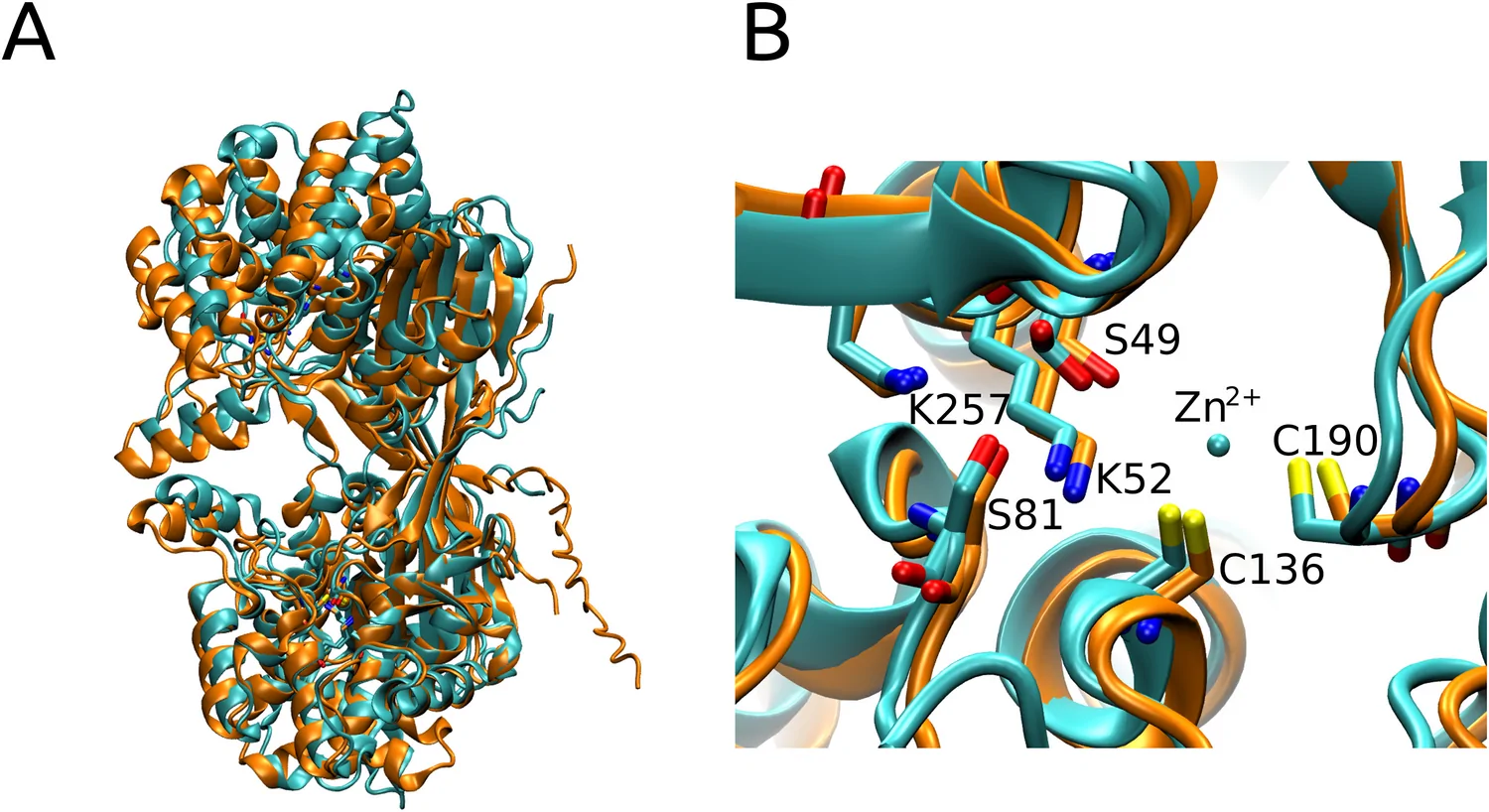
**A** Aligned structures of dimeric l-asparaginases. Experimentally resolved structure of l-asparaginase V (AAF00929.1, PDB ID 7OU1) from *R. etli* (cyan) and ASN339Pt iso 1 predicted by AlphaFold3 (orange). The unstructured chain on the right side is the N-terminal histidine tag and linker, which is only present in ASN339Pt iso 1. **B** Detail of the active site of one subunit (same colorings). Catalytic residues S49 and K52, substrate binding residues: S81 and K257, numbering applies to ASN339Pt iso 1 (without histidine tag sequence). The zinc cation is of ReAV structure 7OU1 and is bound by C135 and C189 residues, which correspond to C136 and C190 in ASN339Pt iso 1

We compared the predicted structure of the ASN339Pt iso1 dimer to the experimentally resolved structure of l-asparaginase V from *Rhizobium etli* (AAF00929.1, PDB ID 7OU1, Loch et al. 2021) as it was one of the most similar molecules according to Blast search, with high resolution (1.65 Å) experimentally resolved crystal structure in Protein Data Bank. This finding was further supported by the fact that the amino acid sequence of ASN339Pt iso 1 contains motifs typical for l-asparaginases classified in class 3 (according to classification proposed by Da Silva et al. (2022), respectively, *Rhizobium etli*-type l-asparaginases (historical classification). These motifs are R-S-X_2_-K-P/A–X-Q–A-L (R-S–S-S-K-P–V-Q–A-L in the case of ASN339Pt iso 1) and N/G-C-S-G/A-K-H–X-G/A-M/F (N–C-S-G-K-H–L-G-V in the case of ASN339Pt iso 1 differing in the last amino acid residue) (Da Silva et al., 2022). The aligned structures are shown in Fig. 6.

We observed that although the primary sequences of both l-asparaginases in Fig. 6A are not very close, with only about 28% residue-residue identity (Online Resource 4), the secondary and tertiary structures are identical except for a few loops that differ in size. Loch et al. (2021) identified catalytic amino acids S48 and K51, substrate binding amino acids S80 and K263, and zinc-binding amino acids C135 and C189 in the active site of ReAV. From structural alignment (Fig. 6B, Online Resource 4) it can be seen that the residues of serine (S49 and S81), cysteine (C136 and C190), and lysine (K52 and K257) are in the same positions in ASN339Pt iso 1 (numbered without the histidine tag sequence); therefore, they could participate in catalysis.

Some of these residues are also part of the sequential motifs typical for class 3 l-asparaginases as mentioned above. This also suggests that the activity of both enzymes follows the same mechanism. These findings further support the hypothesis that ASN339Pt iso 1 belongs to the same protein family as l-asparaginase V from *R. etli* despite their many primary sequence dissimilarities.

Due to the determination of the role of Lys52 as a probable catalytic amino acid, which may significantly affect the pH optimum of the enzyme (pK_a_ of ε-amino group in proteins is between 9.4 and 11.0) (Loch et al. 2021), the possibility of shifting the optimum pH to lower values using a point mutation was tested. The catalytic Lys52 residue was replaced by histidine (pK_a_ of imidazole group in proteins is between 5.6 and 7.0). Although the protein containing this mutation was successfully produced, there was a complete loss of l-asparaginase activity.

As for zinc, the comparison of 3D structures shows that ASN339Pt iso 1 contains the same amino acids for its binding as ReAV. However, our experiments showed that 10 mM EDTA decreased the activity of ASN339Pt iso 1 only to 83% and the addition of 50 μM Zn^2+^ increased the activity up to 92% (not shown here). It can be concluded that if Zn^2+^ is present in the active site of ASN339Pt iso 1 (similarly to ReAV), it does not play an essential role in the catalytic mechanism of ASN339Pt iso 1.

## Discussion

This work was devoted to one of the two l-asparaginase isozymes (ASN339Pt iso 1) that are produced by the bacterium *Paenibacillus thiaminolyticus*. According to our results, ASN339Pt iso 1 belongs to class 3 l-asparaginases (classification proposed by Da Silva et al., 2022), respectively, *Rhizobium etli*-type l-asparaginases (historical classification) and therefore to the group of l-asparaginases which currently contains the fewest known representatives (Da Silva et al., 2022). To date, enzymes from bacteria and fungi that belong to this group of l-asparaginases have been described (Da Silva et al., 2022), including another l-asparaginase from *Paenibacillus*, specifically from *P. barengoltzii* (Shi et al. 2017).

Based on the results obtained from size exclusion chromatography and computer modelling, it is possible to assume that ASN339Pt iso 1 occurs naturally in the form of a homodimer. This finding also corresponds to the results obtained by Loch et al. (2021) on the oligomeric state of l-asparaginase from *R. etli* (ReAV). However, the oligomeric state may not be uniform for all *Rhizobium etli*-type l-asparaginases, as, for example, the native state of l-asparaginase from *P. barengoltzii* was described as a monomer (Shi et al. 2017). In general, l-asparaginases have been described in various oligomeric states, including monomers, dimers, tetramers, and hexamers (Batool et al. 2016).

In the ReAV structure, one Zn^2+^ ion was confirmed to be localized in the active site (Loch et al. 2021). Later, experiments were performed to elucidate the role of Zn^2+^ in ReAV. Sliwiak et al. (2024) investigated the appropriate concentration of this ion to obtain the highest l-asparaginase activity, and they also created the mutant forms of ReAV in order to disrupt interactions between selected amino acids and the zinc cation (Sliwiak et al. 2024). This work confirmed that the activity of mutant ReAV was affected by the disability to coordinate the zinc cation properly (Sliwiak et al. 2024). Since the 3D structures of ReAV and ASN339Pt iso1 are very similar, it is possible to consider that this zinc cation could also be located in the active site of ASN339Pt iso1. However, when treating this enzyme with EDTA, we found that if Zn^2+^ is present in the active site of ASN339Pt iso1, its removing does not have a decisive effect on its activity.

Given the importance of l-asparaginases for the pharmaceutical and food industries, it is not surprising that there is a growing demand for their ease of production. The most commonly used system for recombinant production is undoubtedly *E. coli*; although, other systems such as *Bacillus subtilis* or *Pichia pastoris* (renamed *Komagataella phaffii*) have also been tested. In several cases, this approach was successful, as comprehensively discussed in the following publications (Barros et al., 2021; Castro et al. 2021; Lefin et al. 2023). In this work, two different production systems (*E. coli* BL21 (DE3) and *E. coli* Lemo21 (DE3)) and two culture media (classical LB medium and autoinduction medium) were tested under several conditions. The best results were achieved using *E. coli* Lemo 21 (DE3) cells either in LB medium with 100 µM rhamnose after 4 h of cultivation (final yield 337 mg L^−1^) or in autoinduction medium with 500 µM rhamnose after overnight cultivation (final yield 335 mg L^−1^). Although yields are comparable in both cases, from an economic point of view, cultivation in LB medium is clearly more advantageous. Taking into account our costs for both Overnight Express™ Instant LB Medium (autoinduction medium) and LB medium (in house prepared from NaCl, yeast extract and tryptone), 1 L of autoinduction medium costs about $143, while the same volume of LB medium costs only approximately $2.

The dependence of the initial reaction rate on the substrate concentration did not follow the classical kinetics described by Michaelis and Menten. Substrate inhibition is rarely reported for l-asparaginases, but it has been observed, for example, in l-asparaginase from *Pyrococcus furiosus* (Bansal et al., 2010). The decrease in the initial reaction rate was observed for concentrations of l-asparagine higher than 20 mmol L^−1^ (*K*_*M*_ for this enzyme is 12 mmol L^−1^) (Bansal et al., 2010). Substrate inhibition was not described for similar l-asparaginase from *R. etli* (AAF00929.1); however, the dependence of the initial reaction rate on substrate concentration was described no more than for 40 mM concentration of l-asparagine in a reaction mixture (Moreno-Enríquez et al. 2012). There are also known cases where the determined decrease in enzyme activity at higher substrate concentrations is caused by compounds which are products of the reaction process. For example, Moreno-Enríquez et al. (2012) referred to the inhibition influence of l-aspartate and NH_4_Cl on the catalytic activity of l-asparaginase from *R. etli* (AAF00929.1; Moreno-Enríquez et al. 2012); however, the concentrations used (5 mM l-aspartate (*K*_*i*_ = 33 mmol L^−1^) or NH_4_Cl (K_i_ = 10 mmol L^−1^) are much higher than the concentration which can be produced during the measurement of the initial reaction rate. In our case, the final concentration of NH_4_^+^ ions (380 µmol L^−1^) in the reaction mixture was designed to match the possible resulting product concentration and to minimize the measurement error caused by the high blank absorbances. No inhibitory effect on the final activity of ASN339Pt iso 1 was observed, as was also the case of 10 mM l-aspartic acid. These results support the theory of substrate inhibition. Interestingly, in this case, it was not possible to apply the commonly used Eq. (1) to describe substrate inhibition, and a more complex model (described in Eq. (2)) considering the binding of multiple substrate molecules had to be used.

Talking about the catalytical properties of ASN339Pt iso 1, substrate specificity is one of the key characteristics of l-asparaginases in view of their potential use. Here, we confirmed that ASN339Pt did not show l-glutaminase, nor urease activity. According to Moreno-Enríquez et al. (2012), *R. etli* asparaginase (ReAV) also does not possess l-glutaminase activity, which could be a typical property of enzymes from this group of l-asparaginases. Since l-asparaginases have been proposed as a potential component of biosensors to detect l-asparagine, their high specificity for this amino acid is a great advantage (Nunes et al. 2021).

The pH range at which l-asparaginases show the highest activity is quite wide and variable and determines the possibilities for their practical use. For example, the BRENDA database lists a range from pH 5.0 (e.g., for *Pseudomonas aeruginosa*) to 9.5 (for *Stenotrophomonas geniculata*) (https://www.brenda-enzymes.org/). In this context, ASN339Pt iso 1 with a pH optimum higher than 10 and 50% retained activity in the pH range 9–11 lies a little out of the group of known enzymes. On the other hand, these results correspond very well to the results obtained for l-asparaginase from *R. etli* (ReAV is most active at pH 10–12) (Loch et al. 2021). The strong decrease in the activity of ASN339Pt iso1 at pH lower than 9 also supports the structure-based hypothesis that uncharged lysine residues present in the active site are necessary for the catalyzed reaction to take place, which was suggested by Loch et al. (2021). The necessity of the catalytic Lys52 was also confirmed by the preparation of the mutant enzyme ASN339Pt iso 1 (K52H) because its replacement with histidine resulted in a complete loss of l-asparaginase activity. Although this amino acid contains a dissociable group that could replace the role of ε-amino group of lysine, its different structure led to disruption of the course of the catalytic reaction. Thus, the mutation confirmed that Lys52 is one of the catalytic amino acids.

The ability of ASN339Pt iso1 to catalyze l-asparagine hydrolysis at high pH opens another application field for this l-asparaginase in the food industry because, as far as we know, there is only one l-asparaginase on the market so far that can be used in cases where it is necessary to reduce the amount of l-asparagine in materials of high pH (PreventASe® XR from DSM, DSM company websites, 2024) to decrease the formation of acrylamide and thus its amount in final products.

As mentioned previously, the new l-asparaginase ASN339Pt iso 1 originating from *P. thiaminolyticus* described in this article belongs, according to its structural features, to the group of *R. etli* l-asparaginases. Interestingly, this l-asparaginase shares only 49.7% sequence similarity with l-asparaginase originating from another member of this genus,* P. barengoltzii*, which was previously isolated and characterized by Shi and co-workers and classified as a member of *R. etli* l-asparaginases (Shi et al. 2017). When comparing their biochemical characteristics, both mentioned l-asparaginases exhibit similar temperature optima around 45 °C. Their pH optima lie in the basic area; however, they differ significantly (8.5 for *P. barengoltzii* vs. 10.3 for *P. thiaminolyticus*). At the same time, l-asparaginase from *P. barengoltzii* shows a slightly higher affinity for l-asparagine as a substrate (*K*_*M*_ values 3.6 mmol L^−1^ vs. 26 mmol L^−1^). Very important is the fact that neither of the two l-asparaginases utilize l-glutamine as a substrate, which is very significant from the perspective of the research on l-asparaginases suitable for biotechnological use.

In conclusion, it can be summarized that characteristics of the new l-asparaginase ASN339Pt iso 1 originating from *P. thiaminolyticus* (especially its high substrate specificity to l-asparagine) and its robustness and simplicity of production make ASN339Pt iso 1 an enzyme with great potential in various practical applications. ASN339Pt iso 1 will be tested for possible use in biosensors determining the amount of l-asparagine in different matrices and as a tool for acrylamide mitigation in food products where activity at high pH is required. If the potential of this enzyme was to be confirmed on an experimental basis, it would be necessary to optimize the production process both in terms of quantity (large-scale production) and yield and in terms of economic feasibility.

## Supplementary Information

Below is the link to the electronic supplementary material.

Supplementary file1 (DOCX 3.29 MB)

## Data Availability

The research used data from public databases. If interested, additional information can be provided by the authors upon request.
