# Supplementary material for: High-yield production and functional analysis of novel rhizobial-type glutaminase-free l-asparaginase from Paenibacillus thiaminolyticus
Source: Folia Microbiol (Praha). 2025 Jul 11;71(4):875–87. doi: 10.1007/s12223-025-01296-y (PMC13541837; doi:10.1007/s12223-025-01296-y)
Supplement: Supplementary file 1 [file 12223_2025_1296_MOESM1_ESM.docx]

**High-Yield Production and Functional Analysis of Novel Rhizobial-type Glutaminase-free l-Asparaginase from *Paenibacillus thiaminolyticus***

**Journal:** Folia Microbiologica

Tomáš Podzimek, Karolína Loužecká, Veronika Urbánková, Jan Beránek, Petra Lipovová, Eva Benešová

Department of Biochemistry and Microbiology, University of Chemistry and Technology, Prague 166 28, Czech Republic

**Corresponding author:** Eva Benešová, Technická 5, 166 28 Prague 6, Czech Republic

[eva.benesova@vscht.cz](mailto:eva.benesova@vscht.cz)

Online resource 1 Nucleotide and amino acid sequence of ASN339Pt iso 1 including the N-terminal histidine tag and Factor Xa cleavage site

Online resource 2 The effect of NaCl on the activity of ASN339Pt iso 1

Online resource 3 Chromatogram after the separation of the proteins by size exclusion chromatography. Separation was performed using column HiPrep™ 16/60 Sephacryl S-100 HR (GE Healthcare) under these conditions: flowrate 0.5 mL min^-1^, 50 mM phosphate (K^+^) buffer pH 8.0. **A)** Separation of standard proteins, peak 1-aldolase (160 kDa), peak 2-amylase (55 kDa), peak 3-lysozyme (14 kDa). **B)** Separation of ASN339Pt iso 1

Online resource 4 Alignment of the sequence of both ASN339Pt iso 1 and *R. etli* ReAV (AAF00929.1). The colors mark catalytical, substrate-binding, and Zn-binding amino acids. Catalytical amino acids: S48 and K51 (*R.e.*), S49 and K52 (*P.t.*), substrate binding AAs: S80 and K263 (*R.e.*), S81 and K257 (*P.t.*), Zn-binding AAs: C135 and C189 (*R.e.*), C136 and C190 (*P.t.*). The alignment was made by BLAST
